# Gamma irradiation to control *Corcyra cephalonica* and the effect on some physical and microbiological qualities of rice

**DOI:** 10.1038/s41598-025-02517-7

**Published:** 2025-05-22

**Authors:** A. Hammad, A. Gabarty, R. A. Zinhoum

**Affiliations:** 1 Radiation Microbiology Research Department, National Center for Radiation Research and Technology (NCRRT), Egyptian Atomic Energy Authority, Cairo, Egypt; 2 Natural Products Research Department, National Center for Radiation Research and Technology (NCRRT), Egyptian Atomic Energy Authority, Cairo, Egypt; 3 Stored Product Pest Department, Plant Protection Research Institute, Agriculture Research Center, Giza, Egypt

**Keywords:** *Corcyra cephalonica*, Gamma irradiation, Rice quality, Entomology, Microbiology

## Abstract

The rice moth, *Corcyra cephalonica*, is one of the most economically serious stored product pests in many parts of the world. The present study aims to use a safe and eco-friendly irradiation method to disinfest rice of the insects and evaluate the effectiveness of irradiation treatments on various development stages of *C. cephalonica* and some quality properties of rice. The irradiation process was achieved in a Co60 - Gamma Chamber (4000 A) using the doses up to 700 Gy of gamma radiation. Radio-tolerant stage, if the prevention of egg hatching of F1 generation is the criterion for measuring the efficacy of irradiation doses. The dose level of 350 Gy was required when 5-day-old pupae were irradiated. The large-scale confirmatory tests were applied to 16,000 pupae of *C. cephalonica* irradiated with 350 Gy, resulting in a confidence level of 80.797% that 99.99% of eggs laid by adult irradiated as late pupae will not hatch. This irradiation dose had no significant adverse effect on rice’s physical and microbiological quality. Thus, an irradiation dose of 350 Gy is required for the disinfestation of the rice moth .

## Introduction

The rice moth, *Corcyra cephalonica* (Stainton): Lepidoptera: Pyralidae, is an economically important stored grain pest in Asia, Africa, North America, and Europe. It is a significant pest of rice but also infests wheat, maize, sorghum, dates, ground nuts, raisins, cotton seeds, coffee beans, spices, cocoa beans, and millet^1^. The larvae of rice moth spin tough silken fibers that web in the kernel, and molted skin and generally cause quantitative and qualitative loss and reduce the germ ability of stored stocks^2^. Such food contamination is of greater economic importance than larvae feeding and weight loss. The rice moth is considered one of widely distributed pests around the world^3^. But until now I think that there is no information about the actual program to combat this pest.

Adults of *C. cephalonica* are short-lived but lay 150—200 eggs per female within a few days after emergence. Eggs are laid on the grains, among grains, in containers, or on any surface near the grains, either singly or in clusters. Eggs have an incubation period of 4–5 days. The first instar, after hatching, moves and feeds on broken grains for some time and then starts spinning a web to join grains. The larval period is 25–35 days in summer and may be extended in winter. Pupation occurs inside an extremely tough, opaque, whitish cocoon surrounded by webbed grains. The pupal period is about 10 days but may extend to 40–50 days over winter. There are 4–6 generations per year^4^.

The traditional control of insect pests in stored products in Egypt, like in other countries, uses fumigants and other chemical insecticides. The use of fumigants leads to a problem of harmful residues in the treated foods and causes resistance to certain insect pests^5,6^. Phytosanitary irradiation (PI) is increasingly used as an alternative to fumigants to disinfest fresh commodities and stored products with quarantine pests^6,7^. The usefulness of PI includes the absence of any residues and insignificant changes in the physicochemical properties or the nutritive value of the treated products. Development of resistance by pest insects to radiation is unknown^8^. It was believed that the disadvantage of the commercial application of quarantine treatment by gamma irradiation is that no acute insect mortality occurs. This issue is fundamental because when inspectors find live quarantine pests from the major phytosanitary treatments, the entire consignment may be rejected or retreated regardless of treatment certification. In this case, the inspectors assume that the treatment was not properly done or commodity re-infested after treatment^9^. Nevertheless, the objective of irradiation is not acute mortality but prevention of development or reproduction, as most commodities do not tolerate the usual dose ranges required to reach acute mortalities (usually ≥ 1 kGy)^10^. Therefore, inhibition of further development must be considered as a measure of the efficacy of phytosanitary irradiation. The Animal and Plant Health Inspection Service (APHIS) and the International Plant Protection Convention (IPPC) approved phytosanitary irradiation treatments for more than 20 insect pest species^11,12^.

The effect of gamma irradiation on rice moths was previously reported by many investigators, such as Abou and El-Sawaf^13^ on the life span of adults, Boshra Ahmed^14^ on the larvae stage, and Farghaly et al.^15^ at the pupae stage. However, before PI treatments can be recommended, more information is needed on the effectiveness of irradiation doses on all developmental stages of insects to determine the most tolerant stage to irradiation^16^. Irradiation is the ideal technology for developing generic treatments because it can effectively control pests at a dose level that does not significantly changes the treated products’ quality and/or nutritive value. Moreover, this technology does not leave residuals or allow any resistance development in insect pests^17–19^.

The main objectives of this investigation are:

1. To evaluate the effect of gamma irradiation on various development stages (eggs, larvae, pupae, adults) of *Corcyra cephalonica*.
2. To determine the most radio-tolerant stage and establish the criterion for phytosanitary efficacy.
3. To confirm the phytosanitary irradiation dose using a large-scale confirmatory test.
4. To evaluate the effect of phytosanitary irradiation dose levels on rice’s physical and microbiological quality.

## Results

**Fig. 1:**
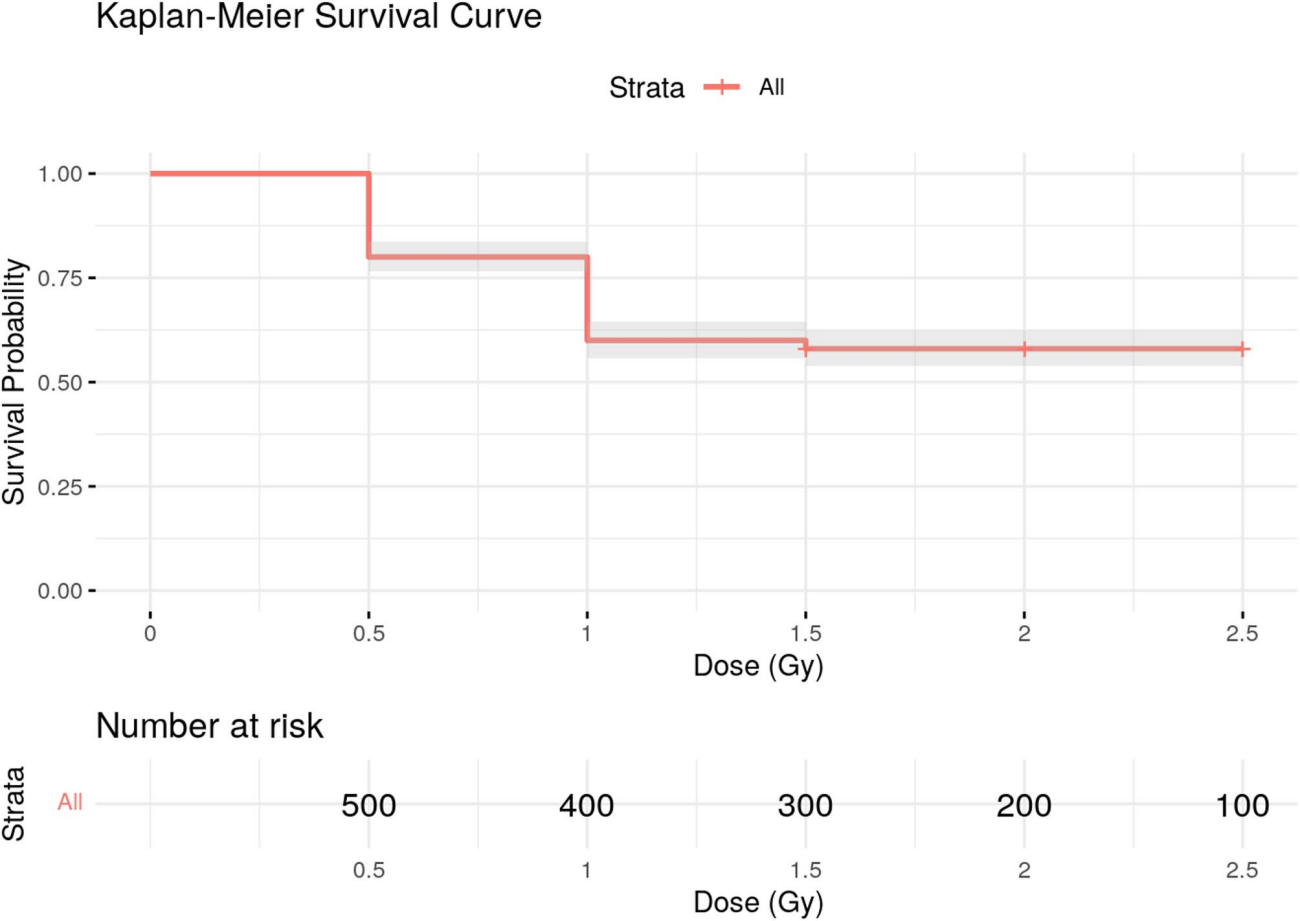
Kaplan–Meier survival analysis of egg hatching of *Corcyra cephalonica*. affected by different doses of gamma irradiation.

The mean percentage of egg hatching and the adult emergence of rice moth decreased with increasing irradiation dose when 3-day-old eggs were irradiated. At the dose level of 200 Gy, 90.6% of adults failed to emerge. No hatched eggs from F1 have been seen at this irradiation dose, indicating that the dose level of 200 Gy prevented F1 egg hatching (prevent hatchability in F1 generation). At the dose level of 250 Gy, 100% of the adults failed to emerge, indicating that this dose induced the non-completion development of the immature stage. However, a higher irradiation dose of 500 Gy completely prevented egg hatching (Table 1). Also. the provided data in Fig. 1 illustrates a clear dose-dependent decline in egg hatching probability following irradiation. Starting from a baseline survival rate of 72.4% at 0 Gy, the percentage of hatched eggs decreases steadily with increasing doses, reaching 40.8% at 350 Gy. This trend highlights the detrimental impact of radiation on egg viability, with the steepest declines occurring at lower doses, as reflected in the Kaplan-Meier survival curve. The curve shows a significant drop in survival probabilities between 0 Gy and 1.0 Gy, followed by a slower rate of decline at higher doses, suggesting a possible threshold or resistance mechanism.

**Table 1:** Effect of gamma radiation on three-day old eggs of *Corcyra cephalonica.*

Dose (Gy)	Hatchability %	No. emerging adult Mean ± SD	Adult emergence %	Fecundity	Hatchability %
				F1 generation	
0	72.4	30.9 ± 5.4^a^	85.4	88 ± 11.4^a^	91.5
50	71.2	29.4 ± 5.4^a^	82.6	73 ± 11.3 ^b^	72.1
100	67.2	19.4 ± 2.3^b^	57.7	37.2 ± 11.9 ^c^	61.8
150	58.4	6.0 ± 1.3^c^	20.5	10.2 ± 3.2 ^d^	31.4
200	55.2	2.6 ± 1.4^c^	9.4	2.6 ± 1.3 ^d^	0.0
250	53.2	0 ± 0	0.0	0 ± 0	0.0
300	48.4	0 ± 0	0.0	0 ± 0	0.0
350	40.8	0 ± 0	0.0	0 ± 0	0.0
400	27.8	0 ± 0	0.0	0 ± 0	0.0
450	16.8	0 ± 0	0.0	0 ± 0	0.0
500	0.0	0 ± 0	0.0	0 ± 0	0.0

Total number of eggs irradiated is 250 in replicates of 50, Duncan Test. Means followed by the same letters are not significantly different at 0.05 level compared between the groups.

The results in Table 2 show that the mortality of the 2nd larval instars increased as the irradiation dose increased and the adult emergence decreased. At the dose level of 150 Gy, the percent of adult emergence resulting from irradiated larvae was 41.7%, and the fecundity was 13 ± 4.1 eggs ∕female and stopped the hatchability of F1 generation. On the other hand, the dose level of 200 Gy prevented adult emergence and the dose level of 350 Gy was required for 100% mortality in the 2nd larval instar.

**Table 2:** Effect of gamma radiation on 2nd larval instars of *Corcyra cephalonica.*

Dose(Gy)	No. larvae dead Mean ± SD	Larval mortality %	No. emerging adult Mean ± SD	Adult emergence %	Fecundity	No. hatched eggs Mean ± SD	Hatchability %
					F1 generation		
0	1.0 ± 0.1^f^	4.0	24 ± 1.1^a^	96	72.6 ± 13^a^	65.2 ± 12.5^a^	89.8
50	9.4 ± 1.5 ^d^	37.6	15.6 ± 1.2^b^	62.4	60.4 ± 9.6 ^a^	35.8 ± 5.8^b^	59.3
100	14.4 ± 2.4 ^c^	57.6	10.6 ± 2.4^c^	42.4	32.2 ± 7.0 ^b^	13 ± 4.1^c^	40.4
150	20.2 ± 1.3^b^	80.8	4.8 ± 1.2^d^	19.2	11.4 ± 1.7^b^	0 ± 0	0.0
200	21.2 ± 0.8^b^	84.8	0 ± 0	0.0	0 ± 0	0 ± 0	0.0
250	21.8 ± 0.8^b^	87.2	0 ± 0	0.0	0 ± 0	0 ± 0	0.0
300	24.0 ± 0.1^a^	96.0	0 ± 0	0.0	0 ± 0	0 ± 0	0.0
350	25 ± 0.0^a^	100	0 ± 0	0.0	0 ± 0	0 ± 0	0.0
400	25 ± 0.0 ^a^	100	0 ± 0	0.0	0 ± 0	0 ± 0	0.0

Total number of 2nd instars irradiated is 125 replicates of 25, Duncan Test. Means followed by the same letters are not significantly different at 0.05 level compared between the groups.

Also, the results in Table 3 indicate that as the irradiation dose increased, the 4th larval instar mortality increased, and the percentage of adult emergence decreased. Irradiating 4th larval instars at the dose level of 250 Gy prevented F1 generation from hatching (prevent F1 egg hatching). Irradiation doses of 550 Gy resulted in 100% 4th larval instar mortality, indicating that the 4th larval instar was more tolerant of radiation treatment than the 2nd larval instar. Consequently, no adults emerged at 550 Gy. When adult emergence of P1 is used as a criterion for measuring the efficacy of irradiation for the control 4th larval instar, an irradiation dose of 550 Gy was required. However, when prevention of F1 egg hatch is used for measuring efficacy, only an irradiation dose of 250 Gy is required.

The 5-day-old pupae were subjected to irradiation doses between 50 and 700 Gy. Table 4 indicates that the average adult emergence from non-irradiated (0.0 Gy) was 93.6%. The lowest irradiation dose (50 Gy) decreased the average adult emergence to 80%. Irradiating pupae at 350 Gy prevented the F1 egg from hatching. No adults emerged when the pupae stage was irradiated at 650 Gy, indicating that this irradiation does prevent pupae development. Thus, the pupae stage was the most tolerant of radiation. When the pupae stage was irradiated, and the prevention of F1 generation from hatch (prevent F1 hatch) was used to measure the effects dose, the irradiation dose level of 350 Gy was sufficient to achieve this purpose (Table 4).

Data in Table 5 show that the percentage of adult mortality increased as the irradiation dose increased. At an irradiation dose of 350 Gy, the adult laid several eggs at P generation. Still, none hatched, indicating the dose level of 350 Gy prevented the hatchability of the F1 generation when an adult stage is irradiated.

**Table 3:** Effect of gamma radiation on 4th larval instars of *Corcyra cephalonica.*

Dose (Gy)	No. larvae dead Mean ± SD	Larval mortality %	No. emerging adult Mean ± SD	Adult emergence %	Fecundity	No. hatched eggs Mean ± SD	Hatchability %
					F1 generation		
0	0.6 ± 0.5^g^	2.4	24.4 ± 1.1^a^	97.6	92.4 ± 5^a^	82.2 ± 9.4^a^	89
50	2.4 ± 1.1^g^	9.6	22.6 ± 1.6^a^	90.4	83.2 ± 11.4^a^	51.6 ± 8.6^b^	62
100	7.0 ± 0.7 ^f^	28	18 ± 1.5^b^	72	68.4 ± 11.9^ab^	24 ± 3.2^c^	35.1
150	9.6 ± 1.5^ef^	38.4	15.4 ± 0.8^c^	61.6	49 ± 8.7^bc^	15 ± 3.2^cd^	30.6
200	10.8 ± 1.3^e^	43.2	14.2 ± 1.6^cd^	56.8	22.4 ± 1.8^cd^	3.4 ± 0.5^d^	15.2
250	11.2 ± 2.3^de^	44.8	13.8 ± 1.3^d^	55.2	5.8 ± 1.6^d^	0 ± 0	0.0
300	13.8 ± 2.7^cd^	55.2	11.2 ± 1.5^de^	44.8	0 ± 0	0 ± 0	0.0
350	14.85 ± 1.5^c^	59.4	10.15 ± 1.1^e^	40.6	0 ± 0	0 ± 0	0.0
400	21.2 ± 1.8 ^b^	84.8	3.8 ± 1.3^f^	15.2	0 ± 0	0 ± 0	0.0
450	21.6 ± 1.1 ^b^	86.4	3.4 ± 0.4^f^	13.6	0 ± 0	0 ± 0	0.0
500	22.4 ± 1.5 ^ab^	89.6	2.6 ± 0.2^f^	10.4	0 ± 0	0 ± 0	0.0
550	25.0 ± 0.0^a^	100	0 ± 0	0.0	0 ± 0	0 ± 0	0.0
600	25.0 ± 0.0 ^a^	100	0 ± 0	0.0	0 ± 0	0 ± 0	0.0
650	25.0 ± 0.0 ^a^	100	0 ± 0	0.0	0 ± 0	0 ± 0	0.0

Total number of 4th instars irradiated is 125 replicates of 25, Duncan Test. Means followed by the same letters are not significantly different at 0.05 level compared between the groups.

**Table 4:** Effect of gamma radiation on the pupal stage of *Corcyra cephalonica*.

Dose (Gy)	No. emerging adult Mean ± SD	Adult emergence %	Fecundity	No. hatched eggs Mean ± SD	Hatchability %
			F1 generation		
0	22.4 ± 1.1^a^	89.6	107.8 ± 9.5^a^	99.8 ± 9.8^a^	92.6
50	20 ± 0.7^ab^	80	77.2 ± 14.2^b^	49.6 ± 4.5^b^	64.2
100	18.2 ± 1.9^bc^	72.8	54.8 ± 19.5^c^	30.2 ± 3.2^c^	55.1
150	15.6 ± 1.8^c^	62.4	50.4 ± 9.4^cd^	20.8 ± 3.9^d^	41.3
200	15.4 ± 0.9^c^	61.6	38.8 ± 8.8^de^	13.8 ± 2.3^e^	35.6
250	11.6 ± 1.1^d^	46.4	32 ± 2.4^ef^	10.8 ± 2.4^e^	33.75
300	11 ± 1.6^de^	44	23.2 ± 5.8^fg^	1.8 ± 0.8 ^f^	7.8
350	9.4 ± 1.7^de^	37.6	20.6 ± 4^fg^	0 ± 0	0.0
400	8.2 ± 0.8^ef^	32.8	11.2 ± 1,6^g^	0 ± 0	0.0
450	6.2 ± 1.8^f^	24.8	0 ± 0	0 ± 0	0.0
500	3.2 ± 1.3^g^	12.8	0 ± 0	0 ± 0	0.0
550	1.8 ± 1.9^g^	7.2	0 ± 0	0 ± 0	0.0
600	0.8 ± 0.8^g^	3.2	0 ± 0	0 ± 0	0.0
650	0 ± 0	0.0	0 ± 0	0 ± 0	0.0
700	0 ± 0	0.0	0 ± 0	0 ± 0	0.0

Total number of pupae irradiated is 125 replicates of 25, DuncanTest. Means followed by the same letters are not significantly different at 0.05 level compared between the groups.

**Table 5:** Effect of gamma radiation on the adult stage of *Corcyra cephalonica.*

Dose (Gy)	No. dead adult Mean ± SD	Adult mortality%	Fecundity	No.hatched eggs Mean ± SD	Hatchability %
			F1 generation		
0	1.4 ± 1.1^e^	5.6	128.6 ± 6.02^a^	116.2 ± 7.04^a^	90.5
50	17.2 ± 1.9^f^	68.8	111 ± 9.9 ^b^	57.8 ± 3.7 ^b^	52
100	19.8 ± 1.4^e^	79.2	69.4 ± 6.9 ^c^	30.2 ± 3.8 ^c^	43.5
150	20.2 ± 1.3^de^	80.8	50.6 ± 16.8^d^	19.2 ± 1.8 ^d^	37.9
200	21.8 ± 1.3^cd^	87.2	38.6 ± 6.4^e^	13.8 ± 2.7 ^e^	35.8
250	22 ± 2.2^bcd^	88	24.8 ± 5^f^	7.2 ± 0.8 ^f^	29
300	22.6 ± 1.1^bc^	90.4	18.4 ± 2.7 ^fg^	1 ± 1.4 ^g^	5.4
350	23.8 ± 1.1^ab^	95.2	8.4 ± 1.1^g^	0 ± 0	0.0
400	25 ± 0.0^a^	100	0 ± 0	0 ± 0	0.0

Total number of adult irradiated is 125 replicates of 25, Duncan Test. Means followed by the same letters are not significantly different at 0.05 level compared between the groups.

In the large-scale validation tests, a radiation dose of 350 Gy applied to 16,500 *C. cephalonica* pupae resulted in zero hatchability percentage (no reproduction) at F1 generation, indicating that this dose is sufficient to control *C. cephalonica* and provide quarantine security. Assuming a required efficacy of 99.99%, C_=_ 1 ^_^ (1 ^_^ 0.0001)^16,500]^, our confidence level was 80.797%. In the non-irradiated control, 500 pupae of C. cephalonica produced an average of 1,820 hatching larvae (Table 6).

**Table 6:** Large-scale confirmatory tests irradiating pupal stage of *Corcyra cephalonica.*

Dose (Gy)		No. replicates	No. irradiated pupae	No. of eggs	No. of hatching larvae
Average measured dose	Target dose				
340–361	350	30	16,500	49	0
	Control	5	500	1,965	1,820

The effect of phytosanitary irradiation dose level (350 Gy) on hulled rice’s color, cooking, and microbiological quality was investigated. The data in Table 7 indicate that there was no significant difference among all color parameters between non-irradiated (0.0 Gy) control rice samples and those irradiated at 350 Gy, indicating that this phytosanitary irradiation dose did not affect the color of hulled rice. Table 8 shows that there was no difference between non-irradiated (0.0 Gy) and irradiated (350 Gy) rice samples in cooking time and the percentage of water absorption at zero time of storage. However, an irradiation dose of 350 Gy increased the volume of irradiated cooked rice by 5.8%. Also, this irradiation dose very slightly increased the weight of cooked rice. After storing non-irradiated and irradiated hulled rice for 3 months, cooking time increased from 15 min to 17 min. Furthermore, the percentage of water absorption and volume decreased. The decrease in irradiated samples was less than that of non-irradiated samples. However, the weight of the cooked rice samples increased, and the increase in irradiated samples was higher than that of non-irradiated ones. Data in Table 9 revealed that the bioburden of raw hulled rice (control samples) was high. Irradiation at 350 Gy very slightly reduced TBC, TM&Y, and coliform counts. The count of *E.coli* in control non-irradiated and irradiated samples was below the detectable level, which is considered negligible. During storage, there was a slight increase in TBC and TM&Y and a slight decrease in coliform bacteria. E. coli was still below the detectable level even after storage for 3 months.

**Table 7:** Effect of 350 gy on the color of hulled rice.

Treatment	Red	Green	Blue	Hue	Sat	Lum
Control	254 ± 0.0	254 ± 0.0	234 ± 2.6	39 ± 0.0	210 ± 0.0	232.7 ± 3.2
Irradiated	254 ± 0.0	253.7 ± 0.6	234 ± 2.6	39 ± 0.0	210 ± 0.0	230.7 ± 1.5

^*^The mean difference is significant at the 0.05 level compared with the control.

**Table 8:** Effect of 350 gy on the cooking parameters of hulled rice.

Parameters	Zero time		3 months storage	
	Non-irradiated	Irradiated	Non-irradiated	Irradiated
Cooking time (min)	15	15	17	17
Water absorption (%)	300	300	200	225
Volume increasing (% )	320	340	215	230
Weight increasing (gm)	241.80	243.58	278.2	325

**Table 9:** Effect of 350 Gyon the microbial quality of hulled rice (cfu/g).

Microorganisms (Log)	Zero time		3 months storage	
	Non-irradiated	Irradiated	Non-irradiated	Irradiated
Total bacterial counts	2.9 × 10^6^	2.8 × 10^6^	2.5 × 10^6^	1.0 × 10^6^
Total molds and yeast	2.55 × 10^4^	2.47 × 10^4^	1.7 × 10^4^	1.4 × 10^4^
Total coliform bacteria	93	75	75	43
*E. coli*	< 10	< 10	< 10	< 10

## Discussion

Our results documented the effect of gamma radiation on the developmental stages of the rice moth *C. cephalonica*, 3-day-old eggs, 2nd and 4th instar larvae, 5-day-old pupae, and 1-2-day-old adults.

- When 3-day-old eggs were irradiated, the dose level of 250 Gy prevented adult emergence, and at the dose level of 200 Gy, the hatchability percentage was zero at F1 generation (Table 1).
- When 2nd instar larvae were irradiated, the dose level of 200 Gy prevented adult emergence, and at the lowest dose level of 150 Gy, the hatchability percentage was zero at the F1 generation (Table 2).
- When 4th instar larvae were irradiated, the dose level of 550 Gy prevented adult emergence, and at the dose level of 250 Gy, the hatchability percentage was zero at F1 generation. The 4th larval instar was more tolerant of radiation treatment than the 2nd (Table 3).
- When 5-day-old pupae were irradiated, the dose level of 650 Gy prevented adult emergence, and at the dose level of 350 Gy, the hatchability percentage was zero at the F1 generation (Table 4).
- When 1–2 days old adults were irradiated, a dose level of 350 Gy was required to prevent hatchability at F1 generation (Table 5).

According to our results, we found that the most tolerant stage to gamma radiation was the pupal stage, and to prevent hatchability at F1 generation, a dose level of 350 Gy was required. However, a higher irradiation dose (650 Gy) is required to stop the adult emergence of the rice moth *C. cephalonica*. Therefore, an irradiation dose of 350 Gy was proposed for large-scale tests to confirm the efficiency of this applied dose for controlling *C. cephalonica* in rice. Hallman et al.^20^ reported that the measure of the efficacy of phytosanitary irradiation is to prevent F1 generation from hatching (prevent F1 egg hatching) when late pupae are irradiated. Previous results in the literature on *C. cephalonica* must be consistent with the proposed irradiation dose to control this insect. Loaharanu^21^ reported that irradiation of *C. cephalonica* eggs at 100 Gy resulted in 100% mortality of hatched larvae in 14 days and 100% mortality of 1–7 days-old pupae. Huque^22^ reported that a female rice moth, *C. cephalonica*, could be sterilized with as little as 100 Gy. Hoedaya et al.^23^ irradiated weevil rice in stored rice and found that 99% were killed at 200 Gy after three weeks. Tilton^24^ reported that the dose of 500 Gy would control virtually all stored product pests by preventing completion of reproduction or adult emergence instead of providing adult mortality. Sehgal and Chand^25^ reported that the irradiation dose of 205 Gy prevented the *C. cephalonica* F1 egg from hatching. Etman et al.^26^ found that an irradiation dose of 500 Gy prevented *C. cephalonica* F1 egg hatching.

Hallman^27^ reported that some lepidopterous pests and most mites required about 300 Gy to control. Abdalla^28^ irradiated full-grown pupae of the rice moth, *C. cephalonica*, at dose levels of 350 and 700 Gy and found that adult emergence was greatly affected. Ignatowicz (2004) irradiated immature (eggs, larvae, and pupae) of *C. cephalonica* at 80 and 100 Gy and observed no development to the adult stage. Farghaly et al.^15^ tested three doses (150, 300, and 450 Gy) of gamma radiation against full-grown pupae of *C. cephalonica*. They found that the dosage level of 450 Gy was the most effective, completely preventing the laid eggs from hatching. The percent of both pupation and adult emergence decreased as the irradiation dose increased. According to previous studies, the irradiation dose required to control *C. cephalonica* ranged from 100 to 650 Gy^15,29,30^.

Our results revealed that an irradiation dose of 350 Gy was sufficient for preventing *C. cephalonica* from completion of development. This irradiation dose is within the reported range (100–650 Gy) of previous studies^28–30^. Therefore, this irradiation dose was proposed to perform large-scale tests to confirm the efficiency of 350 Gy as a phytosanitary treatment. The data in Table 6 indicated that 350 Gy irradiation dose applied to 16,500 pupae resulted in no hatchability of F1 generation (prevent F1 hatch) with a confidence level of 80.797%. Thus, when measuring the efficacy of irradiation dose based on preventing F1 generation from the hatch, the dose of 350 Gy will be sufficient to provide quarantine security. Hallman et al.^20^ reported that the efficiency measure for phytosanitary irradiation is to prevent hatchability of F1 generation resulting from irradiated pupae as a most tolerant stage, which agrees with our study. Early in (1978)^25^ reported that an irradiation dose of 205 Gy applied to late pupae of *C. cephalonica* prevented F1 egg hatch. Etaman et al.^26^ reported that an irradiation dose of 500 Gy applied to male pupae of *C. cephalonica* resulted in no hatching at F1 generation. Farghaly et al.^15^ found that a gamma radiation dose of 450 Gy applied to full-grown male and female pupae of *C. cephalonica* completely prevented the laid eggs from hatching.

Rice (*Oryza sativa* L.) is one of the most essential foods for more than half of the world’s population^31^. It is stored as dry seeds and forms an enormous reserve of food. However, significant quantities of rice seeds are lost due to insect attacks. The rice moth (C. *cephalonica* ) is the most economically important insect. Irradiation treatments as a safe alternative to harmful chemical insecticides are increasingly used to control insects in stored products and fresh horticulture or regulated pests^20,27,32,33^. Developing this safe and friendly technique involves considering the effect of phytosanitary irradiation dose levels on the quality properties of the treated products.

Thus, in the present study, we investigate the effect of phytosanitary irradiation dose 350 Gy as an efficient dose for controlling *C. cephalonica* on hulled rice quality (color, cooking, and some microbiological aspects). Table 7 revealed no difference among color parameters between non-irradiated (0.0 Gy) and irradiated (350 Gy) hulled rice samples, as all color parameters of irradiated samples were almost the same as those of control. Ocloo et al.^34^ irradiated cowpea seeds with gamma irradiation doses of 250,500,750 and 1000 Gy and found that the physical parameters studied were not significantly (*p* ≥ 0.05) affected by these irradiation doses. They also found no significant (*p* ≥ 0.05) effect of irradiation on sensory attributes like flavor, taste, softness, and color of the treated samples. Aylangan et al.^35^ found that irradiation doses of 250, 500, and 1000 Gy did not significantly affect the levels of riboflavin and thiamin in chickpeas, kidney beans, and green lentils. The testers could not differentiate between irradiated and non-irradiated samples in the sensory evaluation. Also, Hammad et al.^36^ found that all the physical and chemical characteristics of cowpea seeds were non-significantly (*p* ≤ 0.05) affected by the irradiation dose of 650 Gy. Generally, all literature relevant to the effect of irradiation disinfestation doses on the major constituents, nutritive value, and organoleptic properties of foods had consistent results that these doses had no obvious effect on the mentioned parameters of irradiated agricultural commodities^35,37–39^ .

Data in Table 8 show the effects of irradiation dose on the cooking parameters of hulled rice. It was evident that irradiation at this dose did not affect the amount of absorbed water; the percentage of absorbed water amount was the same as in the control samples. However, a slight increase in the volume (5.9%) and weight (0.7%) of irradiated cooked rice was recorded. This indicates that 350 Gy irradiation dose, a little bit, improved rice quality. Wu et al.^31^ suggested that using gamma irradiation up to 1 kGy is promising to improve rice eating or cooking quality. During storage for 3 months, the cooking time of non-irradiated and irradiated rice samples was increased from 15 to 17 min. Moreover, the other parameters decreased. This indicates that the storage period affects rice quality rather than the irradiation doses.

Table 9 shows that the initial microbial count of rice was high, indicating a high level of contamination. This high level of contamination could be attributed to the high natural microflora of the rice as well as general conditions during their harvesting, drying, and handling. Irradiation dose of 350 Gy very slightly reduced TBC and TM&Y. Coliform bacteria count was 93 and 75 CFU/g in control and irradiated samples, respectively, indicating a slight effect. *E. coli* was less than the detectable level in both control and irradiated samples. Many investigators reported that irradiation doses used for insect disinfection food slightly reduced the microbial counts of quality attributes^40,41^. AL-Farisi et al.^42^ found that the log of initial TBC of the Fard date was high (4.95), and EBI at 0.5, 1.0, and 2.0 kGy slightly reduced this log count to 4.8, 4.7, and 4.65, respectively. However, several studies indicated the great effectiveness of higher irradiation doses for reducing microbial counts of food to a great extent and eliminating foodborne pathogens. During storage for 3 months, the microbiological count slightly decreased, possibly due to desiccation.

## Conclusion

Tolerance to irradiation increased as the age of any stage increased. 5-day- old pupae were the most tolerant of immature stage. An irradiation dose 350 Gy can be used as a safe phytosanitary treatment to control *Corcyra cephalonica* in rice.

## Methods

### Irradiation process

All irradiation processes were carried out in the central position of the Indian ^60^Co Gamma Chamber (4000 A) at the National Center for Radiation Research and Technology, Nasr City, Cairo, Egypt. The average dose rate in the center of the gamma cell was 1.226 kGy/h. Alanine dosimeters (traceable to the National Physical Laboratory, U.K.) were used to calibrate the gamma cell at the central position and measure absorbed doses in the samples. Detailed dose mapping was conducted by the Department of Radiation Protection and Dosimeters according to Egyptian Standards. The actual doses are equal to the target doses (central doses) ± 3% for the case of rice and insect irradiation, based on alanine dosimetry analyses.

### Laboratory rearing technique

*Corcyra cephalonica* culture was obtained from the Entomology Laboratory at Plant Protection Research Institute, Dokki, Giza, Egypt. The experiment was conducted from 1 January 2017 to 15 June 2018 at the Natural Control Laboratory at the National Center for Radiation Research and Technology & Entomology Laboratory at the Plant Protection Research Institute. The larvae were reared on an artificial diet comprising 500 g wheat bran, 500 g flour maize, 250 ml glycerol, 125 g yeast, 250 ml natural honey, and 250 g milk powder^43^. The emerging moths were introduced into chimney glass cages to lay eggs. It had two openings, one at the top with a dimension of (5.5٭5) covered with gauze after placing moths inside the cage, and the other at the bottom with a dimension of (6.5٭7) covered with wide holes gauze that allowed passage of eggs only. A Petri dish was put under the cage to collect eggs passed through the gauze. Newly laid eggs were collected daily from a Petri dish (90 mm 15* mm). All insect stages were reared in an incubator at 30 ± 20 C and 65 ± 5% relative humidity (R.H.) in continuous darkness.

### Irradiation of eggs stage

Fifty eggs (three days old) were exposed to 0, 50, 100, 150, 200, 250, 300, 350, 400, 450, 500, and 550 Gy of gamma radiation (5 replicates for each treatment were used). The number of hatching larvae in each replicate was counted and transferred to rearing media. The hatchability and adult emergence percentages were estimated. Five separated couples (each couple consisting of a male and female) resulting from each irradiated dose were introduced into chimney glass cages to lay eggs. Newly laid eggs were collected and counted daily from the Petri dish. The hatchability percentage for the F1 generation was estimated^30^.

### Irradiation of larval stage

The 2nd instar larvae (10 days old) were exposed to 0, 50, 100, 150, 200, 250, 300, 350, 400, 450, 500, and 550 Gy of gamma radiation. Also, the 4th larval instars (22 days old) were exposed to 0, 50, 100, 150, 200, 250, 300, 350, 400, 450, 500,550,600, and 650 Gy (5 replicates of each treatment were used). There were 25 larvae in each replicate. The irradiated larvae in each treatment were transferred to rearing media. The number of dead larvae was counted after 7 days. The larval mortality and adult emergency were estimated. Five separated couples (each couple consisting of a male and female) resulting from each irradiated dose were introduced into chimney glass cages to lay eggs. Newly laid eggs were collected and counted daily from the Petri dish. The hatchability percentage for the F1 generation was estimated^30^.

### Irradiation of pupal stage

Twenty-five pupae (5 days old) were exposed to 0, 50, 100, 150, 200, 250, 300, 350, 400, 450, and 500 to 700 Gy of gamma radiation (5 replicates of each treatment were used). The numbers of emerging adults and adult emergence percentages were estimated. Five separated couples (each couple consisting of a male and female) resulting from each irradiated dose were introduced into chimney glass cages to lay eggs. Newly laid eggs were collected and counted daily from the Petri dish. The hatchability percentage for the F1 generation was estimated^30^.

### Irradiation of adult stage

Twenty - five adults (1–2 days old) were exposed to 0, 50, 100, 150, 200, 250, 300, 350, and 400 Gy of gamma radiation (5 replicates of each treatment were used). The number of dead adults was estimated after 5 days. Five separated couples (each couple consisting of a male and female) resulting from each irradiated dose were introduced into chimney glass cages to lay eggs. Newly laid eggs were collected and counted daily from the Petri dish. The hatchability percentage for the F1 generation was estimated^30^.

### Large-scale confirmatory tests

Confirmatory tests were conducted after determining the most tolerant stage that produced zero hatchability in the F1 generation. So, the most tolerant stage was pupae, and the proposed generic dose was 350 Gy. The total number of irradiated 5-day-old pupae was 16,500. Ventilated containers containing 550 male and female pupae were irradiated with 350 Gy (this experiment was repeated 30 times). Five replicates of un-irradiated pupae (each replicate includes 100 male and female pupae) were conducted as a control test. Emerging adults (Irr ♂ × Irr ♀) from each irradiated dose and control test were introduced into chimney glass cages to lay eggs. Newly laid eggs were collected and counted daily from the Petri dish. The number of hatching eggs for F1 generation was estimated.

The level of confidence associated with treating a large number of *C. cephalonica* with zero survival was estimated by the equation: C = 1 – (1 – *Pu*)^n^.

Where *Pu* (0.0001) is the acceptable level of survivorship and n is the number of treated insects^44^.

### Effect of phytosanitary irradiation dose level (350 Gy) on some quality attributes of rice

The de-hulled rice was packed in polyethylene bags (200 g of each) and exposed to 350 Gy of gamma irradiation.

#### Color measurement

The color of de-hulled rice was measured using a Portable Color Analyzer (Model: RGB-1002) equipped with three external phototransistors for measuring values: red (R) – green (G), and blue (B). HSL values: Hue, Saturation (Sat), Luminance (Lum). Three replicates of each sample were used.

#### Cooking

Two hundred grams of irradiated or non-irradiated de-hulled rice were cooked for 15 min in tap water. The percentage of absorbed water, rice volume, and weight increase were determined^45^.

#### Microbiological assessment

Using the standard pour plate technique, the total bacterial count (TBC) and total yeast and mold count (TM&Y) were enumerated on standard plate counts agar and Czapekˊs yeast extract agar media. The inoculated plates for (TBC) were incubated at 30 °C for 3 days, and those for (TM&Y) were incubated at 25 °C for 3–5 days. The count was expressed as a cell-forming unit per gram of sample (CFU/g) by APHA^46^. According to the Charm Operators Manual, total coliform bacteria and *E. coli* were counted on the Charm peel plate EC microbial test (kit code: PP–EC–100k). The inoculated plates were incubated at 35 °C ± 1 for 24 h. At the end of incubation, the plates were observed for colored colonies. Red colonies represent total coliform bacteria, while blue-black colonies represent E. coli. This test has been certified by the AOAC research institute as a performance-tested method #061501.

### Statistical analysis

All statistical analyses were performed at a 5% significance level with the least significant difference using the SPSS (Statistical Package for Social Sciences, ver.17.0) computer program. All measurements’ mean values and standard deviations (SD) were calculated over five replicates. Analysis of variance (One-way ANOVA) followed by post hoc test (Duncan) was performed to analyze the significant difference between all irradiated groups. Kaplan-Meier survival analysis was calculated for Table 1.

## Data Availability

The datasets used and analyzed during the current study are available from the corresponding author upon reasonable request.
